# Winter climate change in the boreal forest—what does it mean for the forest tree seedlings?

**DOI:** 10.1042/EBC20250058

**Published:** 2026-07-30

**Authors:** Johanna Riikonen, Timo Domisch, Françoise Martz

**Affiliations:** 1 Natural Resources Institute Finland, Suonenjoki, Finland; 2 Natural Resources Institute Finland, Joensuu, Finland; 3 Natural Resources Institute Finland, Rovaniemi, Finland

**Keywords:** carbon balance, climate warming, cold acclimation, non-growing season, snow structure

## Abstract

Winter physiological processes are often overlooked in climate change studies of boreal forests, despite their critical role in determining tree survival and carbon balance. Winter climate change is reshaping the environmental constraints that govern boreal forest regeneration, placing increasing pressure on the resilience of tree seedlings. The present mini-review summarises how changing non-growing season temperatures and snow conditions influence the seasonal survival of boreal tree seedlings and the carbon dynamics of both seedlings and soil, with implications for successful forest regeneration. Snow insulates boreal vegetation from extreme cold, regulates light and UV exposure, and shapes spring hydrology. Alterations in snow depth, structure, duration, and melt timing modify soil temperatures, gas exchange, and the snow–soil–root–microbe system, increasing root stress and mortality through deeper frost and hypoxia, ultimately resulting in impaired spring recovery. Winter remains one of the most uncertain parts of the boreal carbon budget, as soils, microbes, and roots continue emitting CO_2_ and climate change is altering these fluxes and their carryover effects. Boreal forests rely on tightly regulated annual cycles in which cold acclimation and frost hardiness are essential for winter survival, yet warming can delay acclimation, increase vulnerability to warm spells, and advance spring phenology, thereby raising frost-damage risk for seedlings. The post-planting resilience of seedlings can be strengthened by targeted silvicultural planning, nursery, and planting practices that better prepare seedlings to withstand increasingly variable and challenging winter conditions. More research is needed on how boreal forest tree seedlings physiologically and phenologically adapt to changing winter conditions.

## Introduction

The boreal forest is one of the world’s largest forest biomes, dominated by cold-tolerant conifers such as pine, spruce, and fir with some other broadleaf species such as poplar and birch. It is typically defined as forest growing in high-latitude regions where freezing temperatures prevail for six to eight months each year [1]. Boreal forests exhibit a tightly regulated annual rhythm, characterized by a short growing season and prolonged winter dormancy. Boreal trees integrate temperature and photoperiod cues to maximize the length of the growing season while avoiding frost damage by preventing premature spring leaf-out and ensuring timely growth cessation in the autumn [2,3]. Although many cellular processes are suppressed when overwintering plants enter dormancy, essential metabolic activity continues to support defence and repair in dormant tissues [4–6].

**Figure 1:**
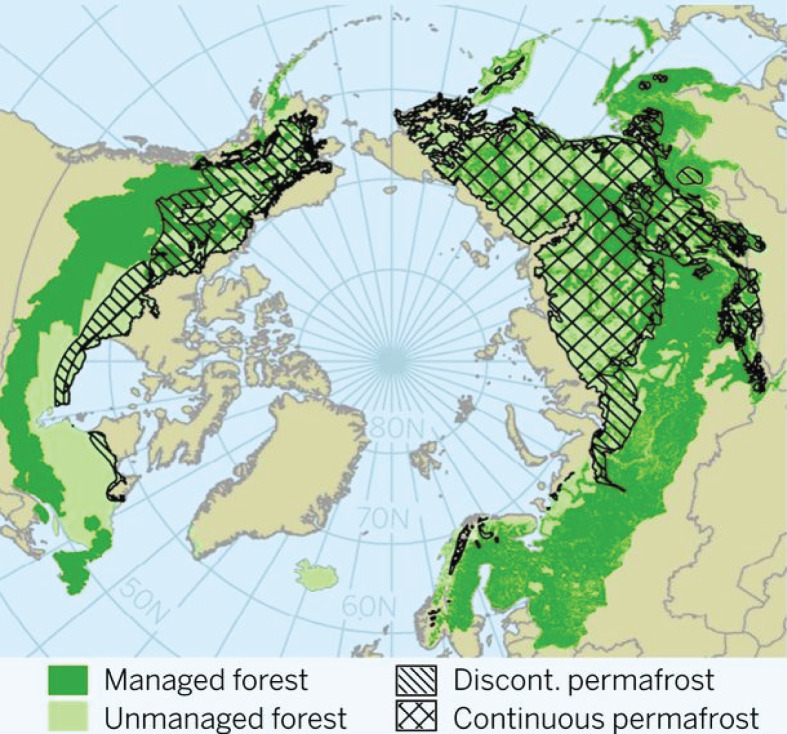
Spatial distribution of managed and unmanaged Boreal forests Figure 1 has been previously published in [15]. Permission to reuse the figure has been granted by the American Association for the Advancement of Science (License No. 6238810008694).

**Figure 2:**
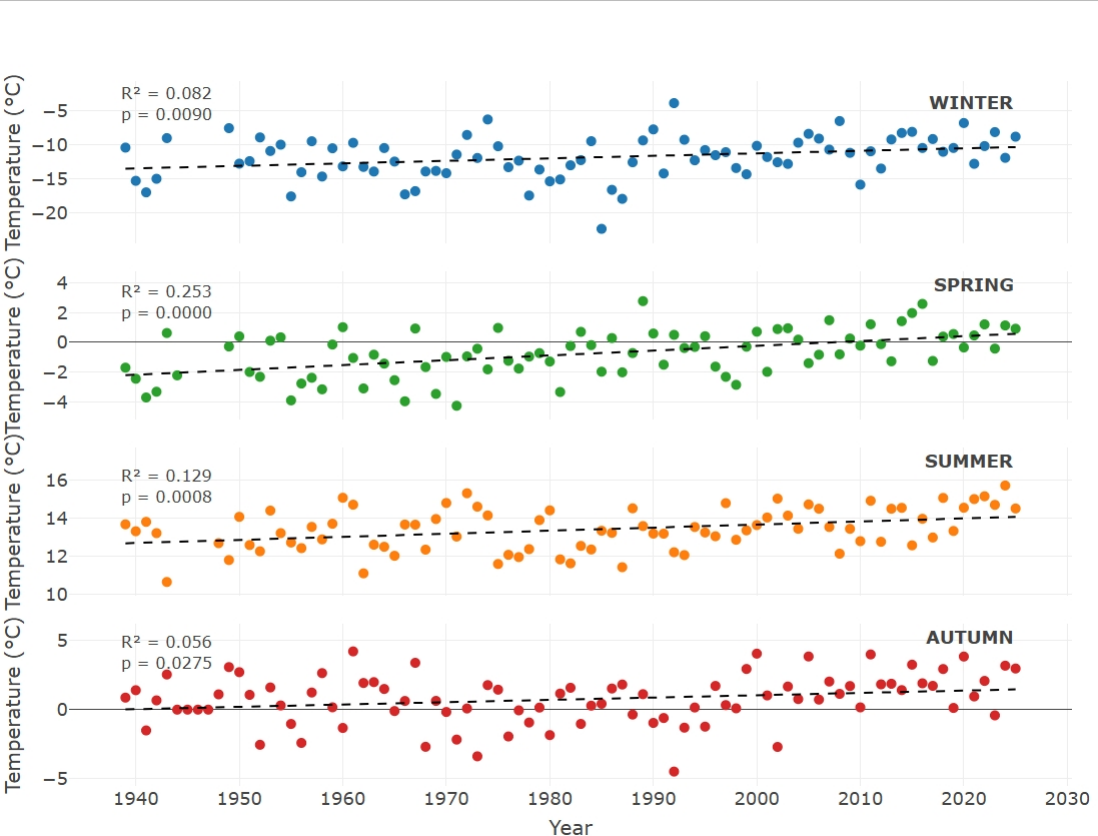
Seasonal warming trends in Rovaniemi Seasonal mean air temperatures for Rovaniemi, Northern Finland, from 1939 to 2025 (winter: Dec–Feb; spring: Mar–May; summer: Jun–Aug; autumn: Sep–Nov; data retrieved from the Finnish Meteorological Institute Open Data service). Linear regression lines were calculated using the base R lm function, and respective *R*^2^ and *P-*values are shown (R 4.5.3).

The boreal forest lies within the circumpolar region (Figure 1) and is particularly exposed to ongoing anthropogenic climate warming, which increasingly challenges the phenological processes of trees. Since 1979, the Arctic (defined as the area north of 66.5°N) has warmed at least three times as fast as the global average [7], with the greatest warming trends during the Oct–May cold season [8]. Similar trends have been measured in the European boreal zone, with the highest increase in temperature during the winter months [9,10]. In northern Finland, analysis of air temperatures since 1940 [11] showed an increase of 0.37°C, 0.32°C, 0.16°C, and 0.17°C per decade during the winter, spring, summer and autumn months, respectively (Figure 2). Constraints that limit plant growth in the boreal forest—such as short, cool growing seasons and low nutrient availability—are therefore changing. The observed shortening of the winter season in northern Fennoscandia [12] is also reflected in vegetation dynamics, with spring recovery advancing by 2.3 days per decade across the boreal evergreen forest zone [13]. Modeling has also revealed a delayed cessation of growth in boreal conifers over the past six decades [14].

In addition to directly impacting tree growth and extending the growing season, higher temperatures affect the duration of snow cover [16] and the properties of the snowpack, due to more frequent warm spells, freeze–thaw (FT) cycles, and rain-on-snow events [17,18]. However, the effect of climate warming on snow cover varies depending on the region.

The impact of climate change on the boreal forest is a very vast topic covered in many reviews at the ecosystem level (e.g. [19–21]), forest level (e.g. [15,22–24]), or both [25]. At the individual-tree level, winter conditions influence the subsequent growing season. In mature trees, higher winter temperatures (December–January) have been associated with reduced tree-ring indices in adult Scots pine the following summer [26]. However, limited data are available regarding seedling overwintering capacities and physiology in mid-winter. The duration and properties of snow cover are particularly important for tree seedlings and understorey forest plants because they remain covered throughout the winter.

The present mini-review focuses on the early phases of tree growth and the abiotic stresses to which seedlings are exposed during the non-growing season (typically September–April), primarily in the north European boreal forest conditions. Good winter survival is critical for seedling establishment, yet limited carbon and nitrogen reserves, small root systems, and soft tissues make seedlings more vulnerable and less resilient to stress than mature trees. The present mini-review synthesizes current knowledge, identifies gaps, and highlights implications for forest management.

**Figure 3:**
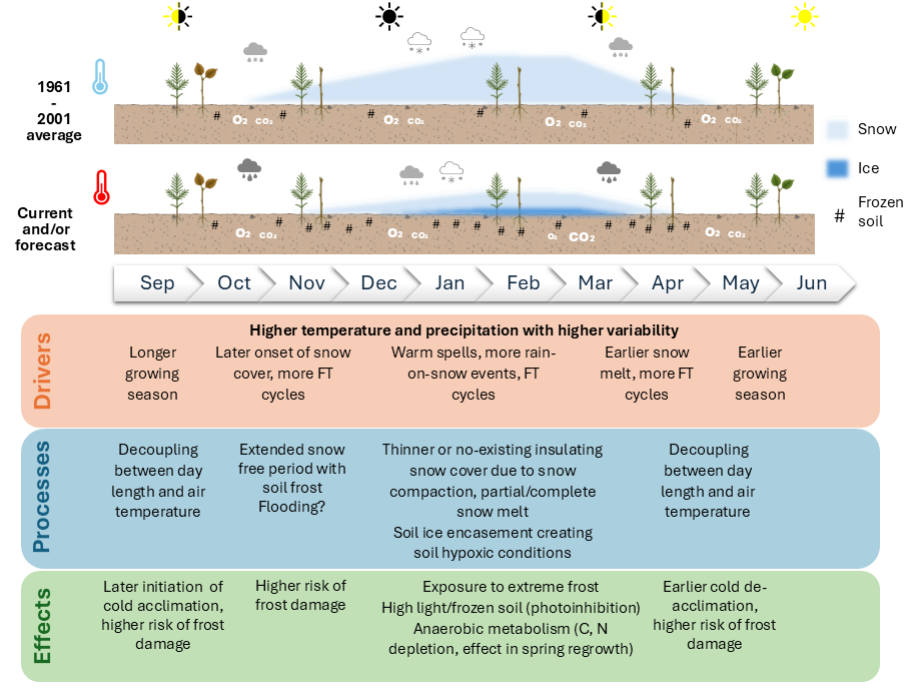
Climate-driven shifts in winter environmental conditions Conceptual illustration of how climate change alters the environmental conditions experienced by plants outside the growing season. The upper panel shows long-term average conditions (1961–2001), characterized by stable snow cover that insulates soil and moderates temperature fluctuations. The lower panel represents current and projected conditions, with reduced and more variable snow cover, increased ice formation, and more frequent FT cycles, leading to colder and more variable soil temperatures. The diagram summarizes key climate drivers, winter processes, and their associated ecophysiological effects. Snow conditions depicted are representative of Finnish Lapland.

To summarize the content of the review, the impacts of climate change on the environmental conditions experienced by plants outside the growing season are presented in Figure 3, including climate drivers, winter processes, and their ecophysiological effects.

## The critical role of the snowpack

Because of its strong insulating properties, snow plays a crucial role in protecting vegetation from extreme freezing temperatures in cold climate ecosystems such as the boreal forest [27]. This insulating effect directly influences the availability of liquid water in the soil, enabling roots, soil fauna, and microorganisms to continue respiring.

Winter soil temperatures can vary widely depending on whether snow cover arrives early, late, or not at all. Reduced or delayed snowpack can lead to lower winter soil temperatures [27–29] and deeper soil frost (Figure 3), which in turn delays soil thawing and the onset of tree growth in spring [29,30]. In some locations, increased winter precipitation falling more often as rain can further decrease soil temperatures [31], known as the paradox of ‘colder soils in a warmer world’ [32,33], even though the number of frost days has declined—and is projected to continue declining—under climate change [12,34].

Although earlier spring snowmelt and later autumn snow onset have been observed with high confidence [35], long-term trends in snow depth remain unclear due to high variability. However, negative trends in snow water equivalent were recorded with high confidence between 1981 and 2016 across both the Eurasian and North American Arctic sectors (regions above 60° N) [36]. Moreover, even after the snow-covered season ends, the timing of spring snowmelt continues to influence ecosystem processes—for example through its effects on water availability, soil moisture, and vegetation phenology.

**Figure 4:**
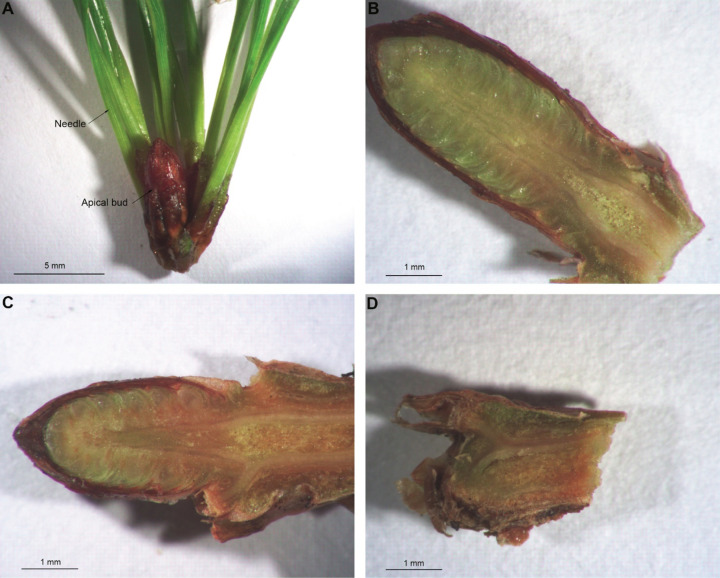
Apical bud damage following snow manipulation Effect of snow manipulation on health of apical buds of Scots pine seedlings in early May in a snow-manipulation experiment in northern Finland. (**A**) Apical bud under stereomicroscope. (**B**) Longitudinally cut of healthy apical bud with no visible damage; (**C**) slightly damaged apical bud; (**D**) heavily damaged apical bud. Republished from [40]. https://doi.org/10.3389/fpls.2022.1050903 Supplementary Figure S3. Bar = 1 mm.

A warmer climate can also change the snow properties, such as increased density, grain size and the fraction of icy and wet snow [37]. Warm spells, more frequent FT cycles, and rain-on-snow events can create ice lenses within the snowpack or form dense, frozen snow layers that lead to soil ice encasement. Because solid ice is nearly impervious to respiratory gases, continued microbial activity and root respiration beneath these layers can cause severe hypoxia or even anoxia, resulting in potentially harmful oxygen-depleted conditions in the soil [28,38] (Figure 3). Ice encasement is most often associated with thaw or rain-on-snow events followed by freezing of the snow cover, but years with ground-ice encasement can be difficult to distinguish from other years when relying on a single climate variable [39]. Norway spruce and Scots pine seedlings subjected to ice encasement in a snow manipulation experiment showed dramatic declines in health. Visual scoring of brown needles indicated a decrease of 47% and 76% in healthy spruce and pine seedlings, respectively, compared to ambient snow conditions [28]. Ice encasement also damaged aboveground structures such as apical buds, particularly in Scots pine seedlings (Figure 4) [40]. Conversely, controlled laboratory experiments show that even dense, frozen snow layers offer better protection against winter desiccation than the complete absence of snow [41,42], highlighting the importance of not only snow amount but also the structure of the snowpack.

## Snow–soil–root interactions

Winter in boreal forests represents a largely dormant period for aboveground plant parts, although belowground processes remain highly active. Boreal tree roots generally exhibit limited cold acclimation capacity compared with aboveground tissues, making them highly dependent on an insulating snow cover to prevent freezing damage [43]. In this regard, even winter can be regarded as a functionally dynamic season during which snow, soil, microbes, and plant roots interact in a tightly coupled system governing temperature regulation, gas exchange, and resource cycling.

Changes in the quantity, timing, and physical properties of the snowpack are reshaping soil thermal regimes and subnivean gas dynamics, with cascading consequences for microbial activity and root physiology. These shifts are particularly critical for seedlings, which possess shallow, limited root systems and minimal storage reserves, making them highly vulnerable to winter stress, and thereby creating a bottleneck for forest regeneration and long-term ecosystem resilience [44,45].

Inadequate distribution of carbohydrates between sources and sinks in seedlings after winters with little or no snow likely reflects disruptions at the soil–root interface. Reduced microbial activity in frozen or oxygen-depleted soils impairs nutrient cycling, while frost damage or hypoxia weakens root sink capacity for assimilates during spring recovery [41]. Domisch et al. further demonstrated that substantial root starch reserves—an essential determinant of spring regrowth capacity—accumulated only in seedlings insulated by snow or an ice layer [41,42]. These processes underline the sensitivity of fine roots, one of the most vulnerable plant organs during winter, to deep soil frost, oxygen limitation, and repeated FT events.

Snow removal experiments in boreal forests consistently demonstrate that reduced snow cover exposes soils to deeper, more prolonged freezing. Such treatments increase fine root mortality by 16%–39% and delay the initiation of spring root growth by 3–6 weeks relative to control conditions [44]. These delays can cascade into the growing season, diminishing nutrient uptake and slowing aboveground recovery. Experimental manipulations with seedlings further confirm that soil freezing reduces root production and longevity and increases mortality [41,42,46] and that frost-related damage is intensified when snow cover is thin or absent [23]. Ultimately, this can have effects on the species composition and cover of understorey vegetation [47]. Several studies indicate that a deeper snow cover may enhance plant performance by providing thermal protection and maintaining more favourable soil conditions [47,48]. Altered winter soil conditions also influence greenhouse gas dynamics. In frozen soils, CO_2_ accumulates, CH_4_ concentrations decline, and N_2_O emissions often peak during early spring when soils are both frozen and waterlogged. These shifts highlight the tight coupling between soil temperature, microbial processes, and root metabolism during winter dormancy and spring thaw. Consequently, physical and hydrological changes to the snowpack affect not only root survival but also broader winter biogeochemical functioning, such as the soil–root–microbe system.

## Non-growing season warming reshape cold and chilling seedling responses

The influence of temperature on the annual growth cycle of boreal tree species has been extensively reviewed [3,44,49–51]. Winter phenology and cold acclimation of aboveground parts have been intensively studied, whereas belowground processes remain much less understood [43,51]. When considering only aboveground organs, two simultaneous physiological processes—development and maintenance of frost hardiness and the induction and maintenance of bud dormancy—determine how well boreal trees can withstand rapid winter climate change.

Increasing autumn and winter temperatures can elevate frost damage risk by disrupting several linked processes in the winter phenology of boreal trees.

Cold acclimation begins after growth cessation, which is triggered by the shortening photoperiod in late summer. In many species, sustained exposure to low autumn temperatures is required to initiate and complete this acclimation process. If this cooling signal is delayed or interrupted by unusually warm conditions, tissues may fail to harden sufficiently before the first freezing events. This risk is increasing under climate warming, as autumn temperatures are rising faster than winter minimum temperatures [52]. Experimental evidence from several tree species supports this pattern of temperature-driven cold acclimation [3,52,50]. However, Noordermeer et al. concluded that autumn warming can delay the downregulation of photosynthesis in Douglas-fir without increasing their susceptibility to freezing damage [53]. Riikonen et al. found that increasing temperature did not elevate the risk of freezing damage in either autumn or spring in three-year-old Norway spruce seedlings [54]. Together, these findings suggest that the impacts of autumn warming on cold acclimation may be species-specific and influenced by growth conditions, underscoring the need for further assessments of freezing risk under future climate scenarios.

Experimental work demonstrates that boreal conifers become increasingly vulnerable to warm spells as winter progresses. Riikonen et al. reported that late autumn warm spells did not reduce frost hardiness in Norway spruce, whereas winter warm spells weakened needle hardiness and increased the likelihood of bud damage; buds were able to regain hardiness afterward, while needles could do so only in early winter [55]. Scots pine and silver birch seedlings were shown to be more vulnerable to fluctuating winter temperatures than Norway spruce [56]. Similarly, Man et al. found that seedlings of several Canadian conifer species exhibited stronger physiological responses to midwinter warm periods and a declining ability to regain frost hardiness from November through March [57]. The reduced ability to reacquire frost hardiness may reflect either depleted carbohydrate reserves required for renewed hardening or irreversible developmental changes during deacclimation [58]. Together, these studies indicate that warm spells followed by freezing conditions can predispose boreal conifer seedlings to winter injury, particularly in midwinter, with species- and tissue-specific differences in vulnerability.

The seedlings’ lack of responsiveness to warm spells in late fall is linked to the buds’ endodormant state, during which growth is inhibited by internal physiological factors and the buds remain largely unresponsive to temperature changes [51,59]. Endodormancy release occurs with the accumulation of chilling temperatures, typically within a range near +5°C. Once the chilling requirement has been fulfilled, ecodormant buds remain in a hardened state until accumulation of post-winter growing degree days allowing growth resumption [49]. According to some studies, climate warming may lead to insufficient chilling accumulation in boreal trees, particularly in broadleaved species [60]. In northern regions such as Finland, however, average winter temperatures still fall clearly within the 0°C–10°C range [61] that effectively contributes to chilling accumulation required for dormancy release. However, as the winter warming is fastest in northern latitudes, southern Finland may face a higher risk in the future.

Warming often accelerates budburst and leaf-out, yet photoperiod can constrain the extent of this change, particularly in northern regions. Therefore, late-flushing forest regeneration material is often recommended to reduce the risk of damage from late spring frosts. However, Aro reported that early-flushing Norway spruce genotypes may experience less spring frost damage, as they move rapidly through their most vulnerable developmental stages and can more quickly readjust their cold tolerance during fluctuating spring temperatures. This suggests a coordinated phenological and physiological strategy that ultimately reduces frost risk [44]. Together, these findings show that spring frost risk cannot be mitigated simply by selecting late-flushing material.

## Non-growing season carbon balance

As growth ceases in autumn, the photosynthetic capacity of conifers is progressively downregulated. Low temperatures interfere with photosynthesis by slowing core metabolic processes such as CO_2_ fixation [52]. At the same time, carbohydrate metabolism shifts toward supporting survival under freezing conditions [5].

Despite these constraints, evergreen conifers remain partially photosynthetically active during winter, and they can even maintain photosynthetic activity beneath the snowpack [62]. Flux-tower measurements show that they can resume measurable carbon assimilation several weeks before snowmelt, thereby contributing to ecosystem-level carbon uptake in late winter and early spring. Deciduous species, in contrast, remain photosynthetically inactive until after snowmelt and therefore have a much shorter active season [63]. Consequently, they must rely on stored starch to fuel regrowth in spring until new leaves reach approximately 50% of their final size and begin to function as net carbon sources [64].

Spring represents an additional stress peak for high-latitude plants. Frequent FT cycles occur at a time when liquid water may not be available from frozen soils and newly exposed shoots may receive extremely high doses of solar radiation. Fresh snow can reflect over 90% of incoming UV radiation onto emerging vegetation [65]. Such conditions commonly induce cold-induced photoinhibition or photo-oxidative damage, reducing photosynthetic capacity for days or weeks and impairing the seedling’s ability to fix CO_2_ and rebuild reserves needed to support new growth [66]. The risk of photooxidative damage is especially high for shade tolerant species like Norway spruce [67].

Despite warmer temperatures extending the potential growing season, shorter day lengths and low autumn radiation continue to constrain photosynthesis and net carbon uptake in northern ecosystems [67]. Moreover, this enhanced non-growing-season photosynthesis becomes increasingly decoupled from growth processes because trees are unable to maintain stem growth under autumn photoperiod and temperature conditions [68]. Instead, the extra photosynthates produced during the non-growing season are primarily stored as non-structural carbohydrates in stems, roots, and branches, where they function as carbon reserves. These reserves can later support spring growth and leaf flush, maintenance and stress tolerance, recovery from environmental stress, and tissue repair or re-hardening after FT cycles [67,68].

In boreal tree species, winter stem photosynthesis occurs particularly in deciduous species with thinner bark and can show measurable activity at temperatures of approximately 0°C–5°C [69]. The process mainly refixes CO_2_ released by stem respiration, thereby reducing carbon loss during the long leafless season. Although low temperatures strongly limit its rate, increased winter light availability due to reduced snow cover, together with rising winter temperatures, may allow somewhat higher, although still low levels of activity. Stem photosynthesis may be most functionally important in late winter before budburst, when it can provide locally produced sugars that support cambial tissues and developing buds prior to leaf emergence (e.g. [70]). Although stem photosynthesis is considered as an important strategy of additional carbon-acquisition [71], its role in overwintering seedling fitness remains largely unknown.

In warming autumns, respiration persists longer than photosynthesis because photosynthesis is strongly constrained by photoperiod, whereas respiration is less photoperiod-dependent and can continue as long as temperatures remain above freezing (e.g. [72]). Respiration continues also under snowpack, and insulation reduces temperature fluctuations [73]. Climate warming extends the time window during which above-ground tissues remain metabolically active, enhancing respiratory CO_2_ release during mild autumn and winter conditions. Even modest increases in winter temperatures can noticeably enhance CO_2_ efflux, intensifying the source-sink phenological mismatch now emerging in boreal conifers under a warming climate [70].

Across boreal regions, soil respiration is the primary component of winter carbon loss, with flux estimates ranging from near zero in deep frozen soils to ∼1.05 g C m^−2^day^−1^ in sites where soils remain unfrozen or near the freezing point. A large latitudinal synthesis shows that soil temperature is the dominant driver of winter CO_2_ efflux. Winter soil respiration typically declines during freezing, but studies show a nonlinear increase in temperature sensitivity when temperatures rise above the freezing point [74]. This demonstrates that even small temperature increases around the freezing point can cause disproportionately large increases in CO_2_ release due to abrupt changes in liquid water and microbial accessibility to substrates. Root activity in winter is usually limited, but roots continue respiring at temperatures well below zero and can significantly contribute to CO_2_ efflux when soils remain partially thawed [75]. Snow-exclusion experiments demonstrate reduced spring water uptake and delayed sap-flow initiation, indicating that winter soil freezing not only suppresses winter carbon fluxes but also creates carry-over effects on spring carbon dynamics through impaired root functioning [23,75].

Winter soil C emissions reflect continued activity of both microbes and roots. Microbial respiration persists wherever liquid water and unfrozen soil remain. Snowpack reduction often increases FT frequency, which can damage fine roots and microbial biomass, thereby altering substrate inputs and enzyme activities. A synthesis of winter warming effects showed that a reduced snow cover can shift soil moisture, pH, nutrient fluxes, and microbial community composition, thus affecting greenhouse gas emissions during winter and spring melt [76].

## Conclusions and future perspectives

Boreal forest functioning during autumn, winter, and early spring is governed by tightly interconnected processes involving snow cover, soil conditions, plant physiology, and carbon dynamics. Snow plays a critical role in insulating soils and sustaining belowground activity, while aboveground survival depends on coordinated cold acclimation and dormancy. Climate warming is disrupting these interactions by altering snow regimes, reducing and destabilizing snow cover, and increasing temperature variability, which exposes soils and seedlings to deeper frost, more frequent FT cycles, and damaging ice encasement.

These changes extend periods of metabolic activity without proportional gains in photosynthesis, leading to greater risks of frost damage, impaired root and microbial function, altered carbon balance, and increased winter carbon losses. The damage is usually only visible in the following growing season, making its causes difficult to identify. Seedlings are especially vulnerable, making winter conditions a key bottleneck for regeneration and long-term ecosystem resilience. Overall, shifts in winter climate are likely to have cascading effects on growth, species composition, and the stability of boreal forest ecosystems.

To sustain successful forest regeneration under these shifting winter conditions and rising temperatures, management efforts must integrate targeted nursery, silvicultural planning, and planting practices.

Using seedlings that are in an appropriate phenological stage and possess sufficient nutrient, water, and carbohydrate reserves enhances their ability to withstand challenging winter conditions. Adaptive silvicultural strategies—including selecting appropriate species and provenances, choosing suitable seedling types, diversifying stand structure, and retaining understorey vegetation—are essential to ensure that newly planted seedlings are well adapted to the microclimatic conditions of the planting site. Planting practices such as avoiding late-autumn plantings—which ensures sufficient root development before winter [77] - and planting of seedlings deep enough, which helps prevent frost heaving and reduces temperature fluctuations around the root collar can further decrease their susceptibility to winter injury [78].

Winter physiological processes are often overlooked in boreal forest climate-change studies, despite their importance for tree survival and carbon balance. More research is needed on maintenance metabolism, use of C and N reserves, root activity under snow, stem photosynthesis, and overwintering under increasingly unstable snow conditions, as well as their long-term ecosystem impacts and implications for forest resilience.

## Summary

- Climatic warming changes the depth and structure of the insulating snow cover and can result in ice layers and increased FT events.
- Adequate snow cover insulates the soil, allowing roots and microbes to maintain low levels of respiration while preventing major carbon losses associated with deep freezing and FT stress.
- Climate warming has the potential to disrupt the cold-hardening processes of forest tree seedlings.
- Warmer winters can expose boreal soils to deeper freezing and soil hypoxia, resulting in severe root damage and weakening seedling survival and spring recovery.
- Winter tree carbon dynamics reflect a balance of downregulated photosynthesis, continued respiration, and limited stem and soil activity, increasingly disrupted by warming-induced source–sink mismatches.

## Supplementary Material

Supplementary Figure S3

## Open Access

Open access for this article is provided via our Subscribe to Open programme, made possible through the support of our subscribing institutions. Learn more here: https://portlandpress.com/pages/open_access_options_and_prices#conditional.

## Abbreviations

FT: freeze–thaw
