# Supplementary material for: Winter climate change in the boreal forest—what does it mean for the forest tree seedlings?
Source: Essays Biochem. 2026 Jul 30;70(1):87–98. doi: 10.1042/EBC20250058 (PMC13424992; doi:10.1042/EBC20250058)
Supplement: Supplementary Figure S3 [file EBC-2025-0058C_supp.pdf]

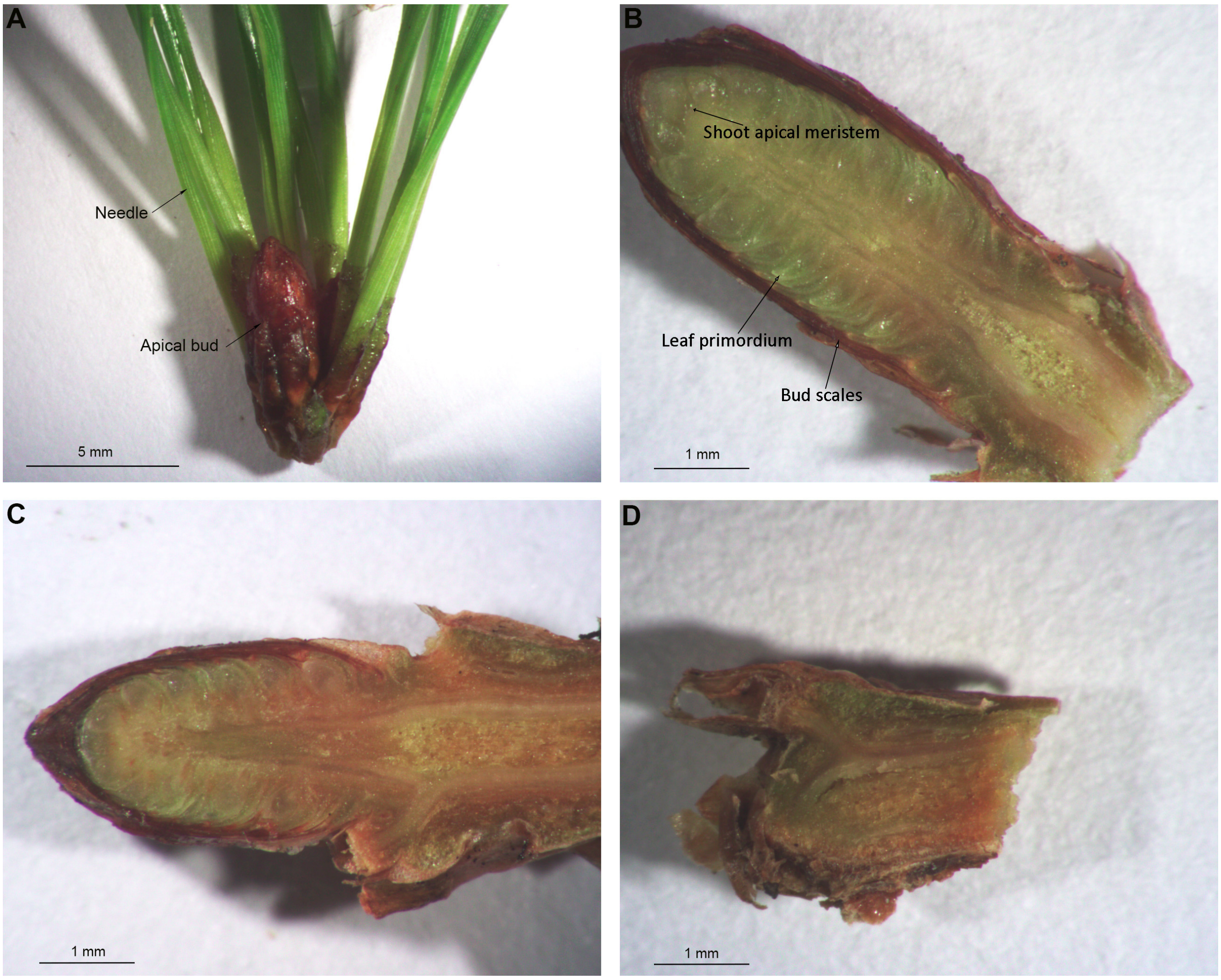

Supplementary Figure 3. Classification of apical buds. On the first sampling date (from May 4 to 7), the inner parts of the longitudinally cut apical buds were examined under a stereomicroscope to evaluate winter damage. Apical buds were classified as healthy, slightly damaged or heavily damaged according to their health. (A) Apical bud under stereomicroscope. (B) Longitudinally cut healthy apical bud with no visible damage. The shoot apical meristem is dormant during winter when it is protected by tightly closed bud scales and the developing leaf primordia. The shoot apical meristem becomes active in the beginning of the growing season, and after the bud scales drop off the leaf primordia develop into new needles. (C) Slightly damaged apical bud. (D) Heavily damaged apical bud.
